# Human RNase H1 Is Associated with Protein P32 and Is Involved in Mitochondrial Pre-rRNA Processing

**DOI:** 10.1371/journal.pone.0071006

**Published:** 2013-08-22

**Authors:** Hongjiang Wu, Hong Sun, Xuehai Liang, Walt F. Lima, Stanley T. Crooke

**Affiliations:** Department of Core Antisense Research, Isis Pharmaceuticals, Inc., Carlsbad, California, United States of America; University of Medicine and Dentistry of New Jersey, United States of America

## Abstract

Mammalian RNase H1 has been implicated in mitochondrial DNA replication and RNA processing and is required for embryonic development. We identified the mitochondrial protein P32 that binds specifically to human RNase H1, but not human RNase H2. P32 binds human RNase H1 via the hybrid-binding domain of the enzyme at an approximately 1∶1 ratio. P32 enhanced the cleavage activity of RNase H1 by reducing the affinity of the enzyme for the heteroduplex substrate and enhancing turnover, but had no effect on the cleavage pattern. RNase H1 and P32 were partially co-localized in mitochondria and reduction of P32 or RNase H1 levels resulted in accumulation of mitochondrial pre ribosomal RNA [12S/16S] in HeLa cells. P32 also co-immunoprecipitated with MRPP1, a mitochondrial RNase P protein required for mitochondrial pre-rRNA processing. The P32-RNase H1 complex was shown to physically interact with mitochondrial DNA and pre-rRNA. These results expand the potential roles for RNase H1 to include assuring proper transcription and processing of guanosine-cytosine rich pre-ribosomal RNA in mitochondria. Further, the results identify P32 as a member of the ‘RNase H1 degradosome’ and the key P32 enhances the enzymatic efficiency of human RNase H1.

## Introduction

Ribonuclease H hydrolyzes the RNA strand in RNA-DNA hybrids [1]. RNase H activity appears to be ubiquitous in most organisms [2]–[8]. Although RNases H constitute a family of proteins with different molecular masses, the nucleolytic activity and substrate requirements appear to be similar for the various isotypes. For example, all RNases H studied to date function as endonucleases, exhibiting limited sequence specificity and requiring divalent cations (*e.g.* Mg^2+^ and Mn^2+^) to produce cleavage products with 5′-phosphate and 3′-hydroxyl termini [8].

Two major types of RNase H exist, namely types 1 and 2, based on sequence conservation and substrate preference [9]. For example, strong amino acid sequence homology was observed for type 1 RNase H (RNase H1) from human, yeast, chicken, *E. coli* and mouse. [10]. Similarly, type 2 RNase H (RNase H2) from human, *Caenorhabditis elegans*, yeast and *E. coli*. exhibit strong sequence homology [11]. Both enzymes have been shown to be expressed ubiquitously in all cells and tissues. RNase H1 enzymes hydrolyze RNA/DNA hybrids containing at least four ribonucleotides [12], while type 2 enzymes can hydrolyze DNA-RNA containing a single ribonucleotide [7], [12]–[14].

Eukaryotic RNases H are larger and more complex than their prokaryotic counterparts. Eukaryotic RNases H2 are composed of three subunits: a catalytic subunit (RNase H2A) that is similar to the monomeric prokaryotic RNase H2, and two other auxiliary subunits (2B and 2C), which are necessary for catalysis by subunit 2A [15]. All eukaryotic RNases H1 have highly conserved regions at their N- and C-termini separated by a spacer region with variable sequences. Human RNase H1 consists of a N-terminal 73-amino acid region homologous to the hybrid-binding domain of the yeast RNase H1, which prefers to bind RNA/DNA duplexes [16]. The C-terminal 151-amino acid catalytic domain is linked by a 62-amino acid spacer region [15]–[19]. Although the N-terminal hybrid-binding domain was shown to be dispensable for RNase H activity, this region is responsible for the enhanced binding affinity of the human enzyme for the heteroduplex substrate and for the strong positional preference of cleavage [18], [20]–[22]. The conserved C-terminal catalytic domain of human RNase H1 shares strong similarity in structure to the well-studied E. coli RNase H1 [10] and the key catalytic and substrate-binding residues required for activity are conserved [10], [17], [23]–[26]. The catalytic domain of the enzyme binds the RNA/DNA duplex via the catalytic site residues and a protrusion composed of predominantly basic amino acids [27], [28]. Catalysis proceeds via a two-metal ion mechanism [29], [30]. The spacer region of RNase H1 is variable in length and is not well conserved in amino acid sequence and has been speculated to interact with additional proteins or complexes in higher organisms [15]. However, the spacer domain is indispensable for human RNase H activity, as demonstrated by deletion-mutational analyses [17].

Several biological roles have been described for eukaryotic RNases H. RNase H2 is inactive as a monomer and accounts for most RNase H activity in the human cells [1], [11], [31]. Mutations in any of the three subunits of the human RNase H2 enzyme can result in Aicardi-Goutieres Syndrome (AGS), a human genetic neurological disorder, resulting from activating the innate immune response [32], [33]. RNase H2 has been suggested to be involved in DNA replication and repair together with PCNA and/or Fen 1 by removing Okazaki fragments and single ribonucleotide(s) which are mis-incorporated by DNA polymerase [7], [34]–[36].

In contrast, eukaryotic RNases H1 enzymes can function as monomers. They have been implicated in mitochondrial DNA replication during mouse development as deletion of RNase H1 in mice arrested development around E8.5 [37]. Human RNase H1, but not H2, has also been shown to play a dominant role in the RNA cleavage activity directed by DNA-like antisense oligonucleotides (ASOs). The effects of over-expression or reduction of human RNase H1 on the potencies of a number of DNA-like ASOs targeting different mRNAs have been determined previously. It was demonstrated that the potency of ASOs positively correlates with the level and activity of RNase H1 in cultured cells [38]. Moreover, over-expression of human RNase H1 in mouse liver increased the potency of a DNA-like ASO targeting Fas mRNA [38].

In the current study, we isolated RNase H1 interacting proteins and identified human P32 as a binding partner of RNase H1 by a classical co-immunoprecipitation coupled to mass spectrometry. We showed that P32 binds RNase H1 at approximately a 1∶1 molecular ratio, enhances the turnover rate of human RNase H1 and reduces the binding affinity of the enzyme for the heteroduplex substrate, presumably by interaction with the N-terminal hybrid binding domain of RNase H1 enzyme. To our knowledge, P32 is the first protein identified in any organism that binds to RNase H1 and alters its enzymatic activity. Finally, we showed that the RNase H1/P32 complex is involved in mitochondrial pre-rRNA processing, consistent with its co-localization in the mitochondria and the fact that P32 interacts with another protein, MRPP1, required for mitochondrial pre-rRNA processing.

## Materials and Methods

### Plasmid and cell lines

The full length human RNase H1, H2, and P32 cDNAs (GenBank accession numbers NM-002936, NM-006397, and NM-001212, respectively) were used to construct the plasmids with N-terminal Flag- or C-terminal HA-tag in pcDNA3.1 vector (Invitrogen) for transient expression or creation of stable cell lines. The N-terminal GST-P32 expression plasmid was constructed in the pGEX 3X vector (GE Healthcare) and the N-terminal His-tag RNase H1 plasmid was in the pET 3C vector (Novegen, EMD Chemicals) for expression and purification of human RNase H1 and P32 proteins. A number of N-terminal His-tagged RNase H1 deletion mutant plasmids were also constructed using PCR and the same vector. All cloned plasmids were confirmed by DNA sequencing. Both HeLa and HEK 293 cells were obtained from ATCC. HeLa cells were used for transient transfection with Effectene reagent (Qiagen) for plasmid and with RNAiMax (Invitrogen) for siRNA, based on manufacturer's instructions. The HEK cells were used for developing stable cell lines with Flag-RNase H1, Flag-RNase H2 and HA-RNase H1 plasmids. The stable lines were selected with 1 mg/ml G-418 (Invitrogen, Carlsbad, CA). All cells were cultured in a 37°C incubator with 5% CO_2_ in DMEM high glucose medium supplemented with 10% FBS.

### Affinity purification of the RNase H1 complex for mass spectrometry

Cell lysates were prepared in immunoprecipitation buffer as previously described [39]. For immunoprecipitation experiments, 1 to 5 mg protein extract was pre-cleared using mouse IgG agarose beads (Sigma St. Louis, MO) for 1 hour at 4°C. The pre-cleared extracts were incubated with anti-Flag or anti-HA beads (15 µl/mg protein, Sigma, St. Louis, MO) for 2 hrs at 4°C, or incubated with human RNase H1 (20 µg/1 mg protein) [38], H2 (40 µg/mg protein) [40], or P32 (10 µg/mg protein) (Santa Cruz Biotechnology) antibodies for 2 h at 4°C, followed by the addition of protein A agarose beads (Sigma, St. Louis, MO) and incubation at 4°C for another 1 h, as described [38], [41]. The immunoprecipitates were washed four times with RIPA buffer (50 mM Tris, PH 7.5; 10 mM MgCl_2_; 150 mM NaCl; 0.5% NP40; and protease inhibitor). The proteins were then eluted either by heating the beads at 95°C in sample buffer or by adding 50 µl of flag or HA peptide (1 µg/µl), followed by the removal of any remaining associated proteins by heating in the sample buffer. The protein samples were subjected to SDS-PAGE and silver-stained with SilverQuest Silver Staining Kit (Invitrogen, Carlsbad, CA). The bands of interest were excised and the gel slices were further destained (Invitrogen, Carlsbad, CA) and proteins identified by mass spectrometric analysis (The Scripps Center for Mass Spectrometry, San Diego, CA).

### Immunofluorescent staining

For immunofluorescence studies, cells grown in Glass Bottom Culture Dishes (MatTek) were washed twice with 1×PBS, fixed with 4% formaldehyde in PBS for 30 min at room temperature, and permeabilized for 5 min using 0.15% Triton X-100 in PBS. Following three washes with 1× PBS, cells were treated with blocking buffer (1 mg/ml BSA in 1×PBS) at RT for 30 min, and incubated with first antibody (1∶50 rabbit anti-RNase H1 antibody or 1∶150 mouse anti-P32 antibody, Santa Cruz Biotechnology) at 4°C for overnight, or at room temperature for 3 hours. After 3 washes with wash buffer (0.1% NP-40 in 1×PBS), cells were incubated with secondary antibodies (1∶200 TRITC labeled donkey anti-rabbit IgG or 1∶300 FITC labeled donkey anti-mouse IgG) (Jackson Immuno Research, West Grove, PA) in blocking buffer at RT for 2 hours, and washed 3 times with wash buffer. Finally, cells were mounted using Prolong Gold anti-fade reagent containing DAPI for nuclear staining (Invitrogen, Carlsbad, CA) and covered. The mitochondrial staining was performed using Mitotracker (Invitrogen, Carlsbad, CA). Images were taken using a confocal microscope (Olympus) and analyzed with Fluoview Ver. 2.0b Viewer (Olympus). To enhance the RNase H1 staining signal, the adenovirus which over-expresses full length human RNase H1 [38] was used to infect HeLa cells (10–200 pfu/cell) 12–18 hours before immunofluorescence staining.

### Expression and purification of recombinant human RNase H1 and P32

For human RNase H1 expression, the plasmid was transfected into *E. Coli* BL21 cells (DE3) (Novagen, EMD Chemicals). The bacteria were grown in LB medium at 30°C. Expression was induced by incubating cells with 0.5 mM IPTG for 4 hours, harvested at an optical density of 0.8 at A600, lysed in 6 M Guanidine lysis buffer and purified with nickel nitrilotriacetic acid agarose (Qiagen, Valencia, CA). For the expression of GST-P32 and control GST protein, the cells were grown in LB at 37°C, induced with 1 mM IPTG for 2 hours, harvested, lysed in B-Per reagent (Thermo Scientific, Waltham, MA) and purified with GST beads (Pharmcia, GE Health, Piscataway, NJ). The purified proteins were further dialyzed into the storage buffer (20 mM Tris PH 7.5, 50 mM NaCl, 30% glycerol, 1 mM DTT and 1 mM PMST) and stored in −20°C or −80°C.

### Enzymatic cleavage assay

The RNA, DNA and 2′-fluoro RNA oligonucleotides were synthesized by IDT DNA Inc. (Coralville, IW) for enzymatic cleavage assay as described previously [17], [20], [42], [43]. Purified oligonucleotides were greater than 98% full-length as determined by capillary gel electrophoresis analysis. The RNA and DNA probes were 5′-end-labeled with [γ-^32^P] ATP using T4 polynucleotide kinase following standard procedures [42]. Labeled RNAs and DNAs were gel-purified. The specific activity of the 5′-labeled RNA was approximately 6000 cpm/fmol.

The sequences of RNA/DNA duplex substrates for RNase H enzymatic assay were Duplex A: 5′GGAUGGUAGCACAAGUGACA (ISIS449680, sense RNA from Apo B gene sequence, NM_000384) and 5′TGTCCTTGTGCTACCATCC (antisense DNA). The non-cleavable Duplex B containing 5′GGAUGGUAGCACAAGUGACA (uniform 2′-fluoro modified sense RNA and 5′TGTCCTTGTGCTACCATCC antisense DNA), was used for gel shift assay and as a competitor for determining enzyme binding affinity.

The ^32^P-labeled RNA/DNA duplex substrates were prepared as described before [20], [42]. For determining the RNase H activity of immunoprecipitated, samples, beads were washed with RNase H reaction buffer (20 mM Tris pH 7.5, 50 mM NaCl, 2 mM MgCl, 5 mM B-mercaptoethanol and 1∶100 dilution of RNase inhibitor, Invitrogen, Carlsbad, CA). 100–200 µl P^32^-labeled RNA/DNA duplex substrates in the reaction buffer was added directly to the IP samples on the beads and incubated at 37°C. A 10 µl aliquot of the reaction mixture was removed at different time points ranging from 1.5 to 60 min and quenched by adding 10 µl stop solution (8 M urea and 120 mM EDTA). The digested RNAs were resolved by denaturing PAGE and the substrate and cleaved products were imaged and quantified on Molecular Dynamics Phosphor Imager (GE). Multiple turnover-kinetic experiments to determine Km, Vmax and Kcat of RNase H1 were analyzed as described previously [20], [42] in the presence and absence of the P32 protein. The binding affinities (dissociation constants, Kd) were also determined by competition analysis as described [20], [42], [44].

### RNA interference

HeLa cells were grown on 10 cm or 96 well plates in DMEM medium supplemented with 10% fetal calf serum (FCS) and 1% penicillin/streptomycin at 37°C in 5% CO_2_ incubator. Sub-confluent cells (∼30–50%) were treated with RNase H1 siRNA (S48357, #4390826, Ambion), P32 siRNA, or MRPP1 siRNA (Santa Cruz Biotechnologies, SC-78547) at 2 or 20 nM final concentration in Opti-MEM medium containing 5 µg/ml RNAiMax (Invitrogen, Carlsbad, CA) for 4 hours, based on the manufacturer′s instruction. Four hours after transfection, medium was replaced with DMEM medium supplemented with 10% FCS and 1% penicillin/streptomycin. Cells were harvested at 24 or 48 hours after transfection and lysed for total RNA.

### qRT-PCR assay

To evaluate the siRNA-mediated reduction of RNase H1 and P32 mRNAs, total RNA was purified from 96 well culture dishes using an RNeasy 96 kit in Bio Robot 3000 (Qiagen, Valencia, CA) according to the manufacturers protocol. The RNA concentration was measured with ribogreen RNA quantification reagent (Molecular Probes, Eugene, Oregon). mRNA levels were analyzed using qRT-PCR. Briefly, total RNA was analyzed in a 50 µl PCR reaction containing 200 nM gene specific PCR primers, 0.2 mM dNTP, 75 nM fluorescently labeled oligonucleotide probe, 1× RT/PCR buffer, 5 mM MgCl2, 2 U Platinum *Taq* DNA Polymerase (Invitrogen), and 8 U ribonuclease inhibitor. Reverse transcription was performed for 30 minutes at 48°C followed by PCR: 40 thermal cycles of 30 second at 94°C and 1 minute at 60°C using an ABI Prism 7700 Sequence Detector (Foster City, CA). The sequences of primer/probe sets for human RNase H1, (accession number NM_002936) forward primer- CCTGTACTTACTGGTGTGGAAAATAGC-3′, reverse primer- CCGTGTGAAAGACGCATCTG-3′, probe- TGCAGGTAGGACCATTGCAGTGATGG-3′; human P32 (accession number NM_001212), Ambion Assay ID HS00241825-m1. Human MRPP1, forward prime – GCTGTGTAGACTGTTGGGTAG-3′, reverse primer– AGCCATGTAACAAAACCCCAG-3′, probe- CCAGCGGGAACCACTATCTCTGC-3′; pre-16S, forward primer– CAAAGCACCCAACTTACACTTAG-3′, reverse primer-GCCAGGTTTCAATTTCTATCGC-3′, probe-CCCCAAACCCACTCCACCTTACTA-3′; pre-ND3, forward primer-CCAATTAACTAGTTTTGACAACATTCAA-3′, reverse primer-CTCGTAAGGGGTGGATTTTTCT-3′, probe-TTTTGACTACCACAACTCAACGGCTACA-3′.

### Western Blots

Whole cell lysates were prepared as described [38] and the protein concentration was measured using Bradford (Bio-Rad Lab, Hercules, CA). The samples were boiled in SDS-sample buffer and separated by SDS-PAGE using 4–20% Tris-glycine gels (Invitrogen, San Diego, CA) under reducing conditions. The proteins were transferred to a PVDF membrane and processed for immunoblotting using affinity purified antibody at 5 μg/ml. The immunoreactive bands were visualized using the enhanced chemiluminescence method (Amersham, Arlington Heights, IL) and analyzed using PhosphorImager Storm 860 (Molecular Dynamics, Sunnyvale, CA), or exposed to X-ray film.

### Northern Blots

Total RNA was isolated from different human cell lines using RNeasy kit (Qiagen, Germany). 5–10 μg of total RNA was separated on a 1.2% agarose/formaldehyde gel and transferred to Hybond-N+ (Amersham, Arlington Heights, IL) followed by UV cross-linking. After 30 minutes pre-hybridization in Rapid-Hyb buffer (GE HealthCare) at 42°C, hybridization was performed using ^32^P-labeled DNA oligonucleotide probe [45] in the same buffer at 42°C for 2 hours. Membranes were washed three times with 2× SSC/0.1%SDS at 42°C, for 20 minutes each time. Hybridization was determined by autoradiography with Phosphor-Imager Storm 860 (Molecular Dynamics, Sunnyvale, CA). The oligonucleotide probes used for northern blots were mitochondrial 12 S rRNA probe (5′-GATGCTTGCATGTGTAATCTTACTA-3′), 16S rRNA probe (5′-TGGCTAAGGTTGTCTGGTAGTAAGGTGGA-3′), human U3 snoRNA probe (5′-ACCACTCAGACCGCGTTCTCTCC-3′) and 7SL RNA probe (5′-CTCAGCCTCCCGAGTAGCTG-3′).

### Gel Shift Assay

Purified human His-tag RNase H1, GST-P32 and GST proteins (0.02–0.05 µg) were incubated for 30 min at 4°C with 100,000 cpm P^32^-labeled non-cleavable RNA/DNA duplex substrate (duplex C) in gel shift buffer (20 mM Tris PH 7.0, 20 mM NaCl, 2 mM MgCl, 5% Glycerol). The reactions were separated in 5% native polyacrylamide tris-glycine gels and the autoradiograms were analyzed using Phosphor-Imager Storm 860 (Molecular Dynamics, Sunnyvale, CA).

### Detection of protein-protein interaction of human RNase H1 and P32

Purified proteins (GST, GST-P32, RNase H1 and RNase H1 deletion mutants DL-1 and DL-2) were used in this assay. 0.1–0.5 µg GST or GST-P32 protein was first linked to 50 µl GST beads (Pharmcia, GE Health, Piscataway, NJ) which were pre-blocked with 0.5 mg/ml BSA in PBS. After washed a couple of times with PBS, different amounts (0.025–2.0 µg/400 µl) of RNase H1 or RNase H1 deletion mutant was then added into the GST beads under various conditions, including either PBS or 50 mM Tris buffer plus 1 mM TCEP and extra NaCl (0–1 M) with pH 6.0–10.0. After incubation at room temperature for 15–20 minutes, unbound protein samples were collected and the GST beads were washed 4–5 times with the same buffer. The protein samples were then eluted from the GST beads using 20 mM Glutathione in a solution containing 100 mM Tris pH 8.0, 100 mM NaCl and 0.1% Triton ×100. The eluted proteins were further subjected to SDS-PAGE along with a titration of known amounts of pure P32, RNase H1 or deletion mutant protein samples, followed by Western blot with anti RNase H1 and P32 Abs and quantified using Phosphor-Imager Storm 860 (Molecular Dynamics, Sunnyvale, CA).

### Immunoprecipitation of MRPP1 and RPL5

Whole HeLa cell lysates (∼200 µg) were incubated for three hours at 4°C with 30 µl protein A beads pre-coated with 5 µg antibodies against MRPP1 (NBP1-83654, Novus) or RPL5 (ab86863, Abcam). After washing 6 times with buffer (50 mM Tris, pH 7.4; 200 mM NaCl; and 0.01% NP-40), co-precipitated proteins were directly analyzed by SDS-PAGE, followed by western analysis to detect the co-isolation of P32 or RNase H1. Alternatively, washed beads were treated with RNase A or DNase I at 30°C for 30 min, and eluted materials were precipitated and analyzed by western to detect the presence of P32 protein.

### Detection of DNA or RNA in affinity purified RNase H1 complex

2–5 mg cell lysate prepared from HA-RNase H1 cells and HA-P32 transiently transfected cells were used for immunoprecipitation with anti-HA beads (Sigma, St. Louis, MO). The IP samples on the beads were washed at least 3 times and treated with or without DNase I digestion (10 unit/ml in TE buffer) (Invitrogen, Carlsbad, CA) at 37°C for 30 min. After washing two more times, the IP samples were subjected to phenol-chloroform extraction. The RNA and DNA from the IP samples were precipitated with Ethanol and re-suspended in water. For RNA preps, the RNA and DNA mixtures were further treated with DNase I followed by phenol-chloroform extraction and ethanol precipitation. The RNA samples were reverse transcribed for 30 minutes at 48°C in 50 µl reaction buffer followed by PCR reaction: 10–40 thermal cycles of 30 s at 94°C and 1 minute at 60°C. The PCR products were analyzed with standard TAE agarose gel. The two Primers of Set A are located on mitochondrial 16 S and 12 S rRNA regions of the pre-rRNA, respectively, and their PCR product (157 bp) covers tRNA^val^ coding region (forward primer 5′-CATGGTAAGTGTACTGGAAAGTGCA, reverse primer 5′-TGGCTAAGGTTGTCTGGTAGTAAGGTGGA). The primers of Set B were located in mitochondrial CO II and ATPase 6 coding regions, respectively, and their PCR product (220 bp) covers tRNA^lys^ region (forward primer 5′-ATAGGGCCCGTATTTACCCTATAG and reverse primer 5′-GGTAGTTTGTGTTTAATATTTTTAGTTGGGTG). The primers of Set C were located in mitochondrial URF3 (ND3) and URF 4L (ND 4L) coding regions, respectively, and their PCR product (227 bp) covers the tRNA^arg^ region (forward primer 5′-ATCATCATCCTAGCCCTAAGTCTGG and reverse primer 5′-GGGAGGATATGAGGTGTGAGCGATATA). Primer sets for PCR amplification of U16 snoRNA were: 5′-CTTGCAATGATGTCGTAATTTGC-3′, sense; and 5′-TCGTCAACCTTCTGTACCAGCTT-3′, antisense. The PCR product was 80 bp long.

## Results

### RNase H1 Interacts with Protein P32

**Figure 1:**
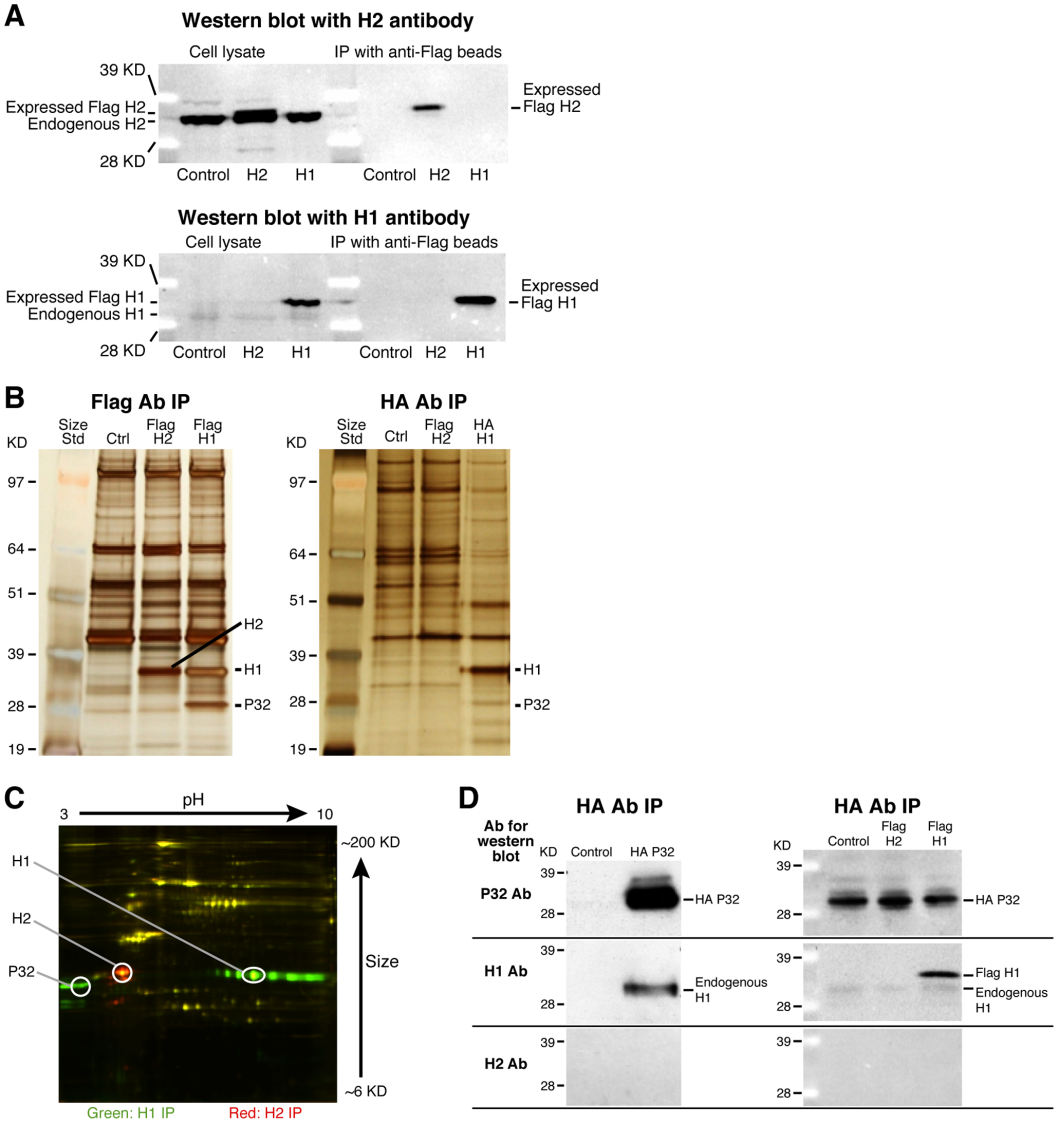
Human RNase H1 is associated with P32. (**A**) Western blot analysis of cell lysates and immunoprecipitated samples show Flag-tagged RNase H1 and H2 expression from cells stably transformed with RNase H1 (H1) or H2 (H2) or wild type (control) HEK cell lines. (**B**) Co-selection of RNase H1 binding proteins by immunoprecipitation. Extracts from cells expressing the Flag-H1, Flag-H2, or HA-H1 cell lines were immunoprecipitated with either anti-Flag or anti-HA antibody. Co-precipitated proteins were resolved by SDS-PAGE, and visualized by silver staining. Protein bands that were different from the co-precipitated proteins from control cells were subjected to mass spectrometry. The protein bands corresponding to the tagged RNase H1, H2 and the co-precipitated P32 proteins are indicated. The size marker was SeeBlue Plus2 Pre-Stained Standard (Invitrogen). (**C**) 2D gel electrophoresis of proteins co-precipitated with Flag-H1 or Flag-H2. About 5 mg cell lysates were prepared for immunoprecipitation with anti-flag beads from cell lines which stably express Flag-H1 or Flag-H2. The immunoprecipitates were washed four times with RIPA buffer and directly sent to Applied Biomics Inc. (San Francisco, CA) for 2D gel electrophoresis coupled with MS analysis. In brief, the co-precipitated proteins from Flag-H1 or Flag-H2 cells were labeled by fluorescent DIGE CyDyers, respectively, followed by 2D gel electrophoresis. The protein image was scanned with a fluorescence detector. The figure illustrates the proteins differentially associated with RNase H1 (green) or H2 (red). The P32 protein was confirmed with mass spectrum from the extracted gel sample. Circled spots were identified as RNase H1, H2 or P32 by mass spectrometric analysis. (**D**) Both endogenous and expressed RNase H1 are co-precipitated with the expressed P32. Left panel: western blots with P32, RNase H1, or H2 antibodies for proteins co-precipitated using anti-HA antibody from extracts of control HeLa cells or cells transfected with HA-P32 expression plasmid. Right panel: western blots for proteins co-selected using anti-HA antibody from extracts of Flag-H1, Flag-H2 stable cell lines and control cells, all of which were transfected with HA-P32 expression plasmid. (**E**) Confirmation of the specific interaction between RNase H1 and P32. RNase H cleavage activity indicates that the P32 co-immunoprecipitated material contains only RNase H1 enzyme activity. Upper panel: Cleavage patterns of human RNase H1 and H2 from IP-coupled enzyme activity assays. Immunoprecipitations were performed with either anti-flag, anti-RNase H1 or anti-H2 antibodies from extracts of Flag-H1, Flag-H2 expressing cells or control cells. The co-precipitated samples were incubated for the indicated times with a ^32^P-labeled RNA/DNA-methoxyethyl (MOE) gapmer duplex and the cleavage products were separated using denaturing gel electrophoresis. The preferred cleavage sites of RNase H1 and H2 are indicated with * or #, respectively. The positions of the preferred cleavage sites in the heteroduplex are shown in the middle panel with the sequences of the RNA substrate (upper strand) and the oligonucleotide (lower strand). The bold nucleotides in the oligonucleotide strand indicate the position of the MOE substitutions. Lower panel: only the RNase H1 enzyme activity was detected in the co-precipitated material from lysates containing tagged P32. Immunoprecipitations were performed with anti-HA antibody from extracts of Flag-H1 or Flag-H2 stable cell lines or control HEK cells, which were all transfected or not transfected with HA-P32 expression plasmid. The precipitated samples were analyzed for cleavage patterns as described above. The position of the cleavage bands relative to the sequence of the cleavage products is shown on the left. A partial alkaline digestion of the same labeled RNA was used as a sequence ladder. The cleavage pattern of purified human RNase H1 is shown at the far right of the lower panel.

To better understand the biological functions of human RNase H1 and its roles in the activity of antisense oligonucleotide-directed substrate RNA degradation, we identified RNase H1-associated proteins by co-immunoprecipitation (co-IP) from cell lines that stably express epitope-tagged human RNase H1 and H2, using antibodies specific to the tags. HEK cell lines were developed to stably express N-terminal Flag-tagged RNase H1 (Flag H1), N-terminal Flag-tagged RNase H2 (Flag H2), or C-terminal HA-tagged RNase H1 (HA H1). Expression of the tagged proteins was confirmed using western analyses with either whole cell lysates or immunoprecipitated materials (Figure 1A). Next, proteins associated with RNase H1 or RNase H2 were co-immunoprecipitated from extracts of cells that stably express the tagged proteins, using either anti-Flag or anti-HA beads (Figure 1B). Co-precipitated proteins were separated by SDS-PAGE and visualized by silver staining. The tagged RNase H1 and H2 proteins were specifically isolated with the corresponding beads, as expected. A protein band (∼28 KDa) was identified that specifically co-precipitated with both Flag H1 and HA H1 RNase H1, but not with Flag H2 (Figure 1B). Mass spectrometric (MS) analysis of the protein band excised from the gel identified the mitochondrial protein, P32 (Gene Bank number NM_001212). The specific co-precipitation of P32 with RNase H1 was also confirmed by 2D gel electrophoresis and MS (Figure 1C). Note that human RNase H1 displays several pIs while RNase H2 displays a single pI of approximately 5. Variable phosphorylation of RNase H1 probably accounts for the multiple PIs.

The specific interaction between Flag H1 and P32 was further confirmed by reciprocal co-immunoprecipitation with transiently expressed HA-tagged P32 (HA-P32) in HeLa cells and in cell lines stably expressing Flag H1 or Flag H2. The endogenous RNase H1, but not H2 was co-precipitated with the HA-P32 in HeLa cells (Figure 1D, left panel). In cells expressing Flag-H1, both the tagged and endogenous RNase H1 proteins were co-precipitated with the HA-P32. As expected, in control HEK cells and Flag H2 cells, only the endogenous RNase H1 was co-precipitated with HA-P32 (Figure 1D, right panel). These results indicate that both the tagged and endogenous RNase H1 but not H2 specifically interact with P32. Note that the endogenous RNase H1 protein is expressed at very low levels compared to Flag H1 expression and is smaller than the flag tagged RNase H1 (Figure 1D, right panel). Consistent with the observations in Figure 1B, neither flag tagged nor endogenous RNase H2 was co-selected with P32. The specific association of P32 with RNase H1 and not H2 was further confirmed by analyzing the cleavage pattern of the P32 co-precipitated materials (Figure 1E) as RNases H1 and H2 have been shown to exhibit different cleavage profiles [40], [46]. The flag-tagged H1 immunoprecipitated with either anti-Flag (Flag Ab) or anti-RNase H1 (H1 Ab) antibody exhibited a cleavage pattern similar to endogenous RNase H1 (Control) precipitated with the H1 Ab. In contrast, a different cleavage pattern was observed for immunoprecipitated RNase H2. Specifically, the preferential cleavage site for RNase H1 (marked with an asterisk) was positioned upstream from the RNase H2 preferred cleavage site (marked with #) (Figure 1E, upper panel). Importantly, the cleavage pattern observed for the HA-P32 co-precipitated material was consistent with the RNase H1 but not RNase H2 cleavage pattern, even in cells over-expressing Flag-H2 (Figure 1E, lower panel). Together, our results confirmed that RNase H1, but not RNase H2, specifically interacts with P32 protein and that the RNase H1 bound to P32 is enzymatically active.

### P32 binds directly to RNase H1 but not the heteroduplex substrate

**Figure 2:**
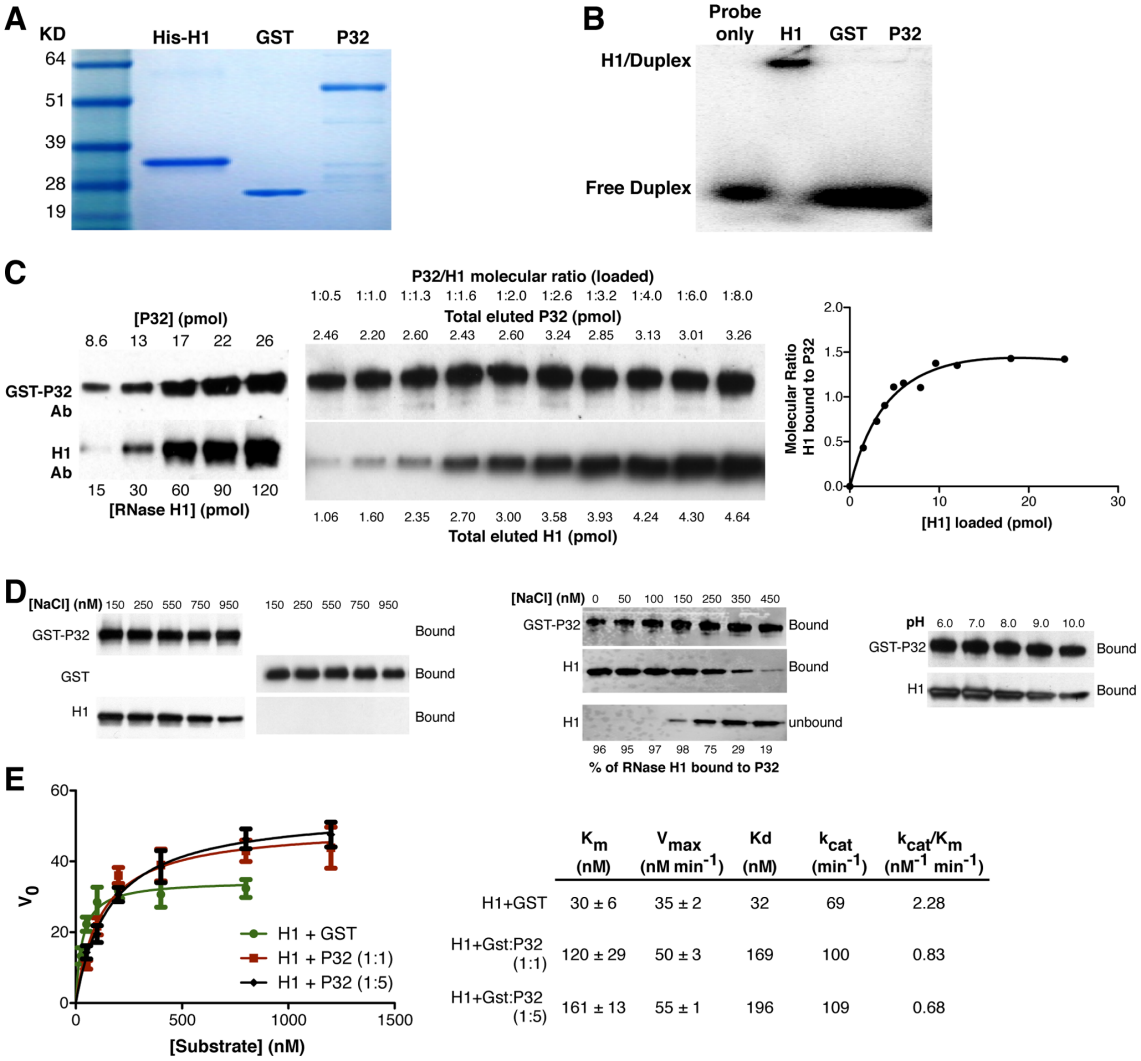
Recombinant P32 binds to recombinant RNase H1, enhances its turnover rate, and reduces the binding affinity of the enzyme for the heteroduplex substrate. (**A**) Coomassie blue staining of the purified human His-H1, GST protein, and GST-P32 proteins separated by SDS-PAGE. The sizes for the standard protein markers are indicated. (**B**) RNase H1 but not P32 appears to bind the heteroduplex substrate. Gel shift assay was performed using 0.4 ug purified RNase H1, GST-P32, or GST proteins incubated at 4°C for 30 min with a non-cleavable heteroduplex containing ^32^P labeled uniformly modified 2′-fluoro RNA annealed to DNA and subjected to native gel electrophoresis. (**C**) The interaction between RNase H1 and P32 appears to be equal molar. A fixed amount of GST-P32 was bound to GST affinity beads and then incubated with increasing amounts of RNase H1. Glutathione (GSH) eluted RNase H1 and P32 were quantified by Western blot as described in the Material and Methods. The amounts of bead-bound P32 and P32-associated RNase H1 were determined by loading known amounts of the respective proteins (left panel). The molecular ratio of bound RNase H1 relative to P32 was calculated and plotted in the right panel. (**D**) The effects of ionic strength on RNase H1/P32 interaction. Left panel: RNase H1 binds GST-P32 but not GST protein. GST or GST-P32 bound to anti-GST beads was incubated with RNase H1 in NaCl concentrations ranging from 0-950 mM as described in the Material and Methods. Middle panel: increasing NaCl concentration inhibits binding of RNase H1 to P32. Both unbound (flow through) and bound (affinity eluted) fractions were collected and the levels of RNase H1 and P32 evaluated by western blot. Right panel: Increasing pH reduced binding of RNase H1 to P32. (**E**) Michaelis-Menten kinetics and binding constants for RNase H1 cleavage of an RNA/DNA duplex in the presence or absence of P32. The K_m_, V_max_, and K_d_ were determined by incubating the Apo B RNA/DNA duplex with RNase H1 plus GST (as control) or RNase H1 plus different amounts of P32 resulting in an H1:P32 ratio = 1∶1 or 1∶5. An uncleavable competitive inhibitor (2′-fluororibonucleotide/DNA) was used to determine the binding to the RNA/DNA duplex, as described in the Material and Methods. The calculated constants are indicated in the right panel. The error bars indicate the standard error from three parallel experiments.

To determine whether P32 interacts directly with the heteroduplex substrate, we incubated purified N-terminal his-tagged RNase H1, a ^32^P labeled uncleavable 2′-Fluroribonucleotide/DNA duplex and either GST-P32 fusion proteins or control GST protein (Figure 2A). The gel shift assay showed that RNase H1, but not P32 or the GST control protein, directly interacted with the substrate duplex (Figure 2B), suggesting that P32 binds to RNase H1 via a protein:protein interaction and not via the heteroduplex. We then determined the molecular ratio of the RNase H1-P32 interaction. Increasing amounts of RNase H1 were added to GST tagged P32 immobilized on anti GST beads and the amount of bound RNase H1 relative to P32 was determined by Western blot analyses (Figure 2C). The amount of RNase H1 bound to the immobilized GST-P32 appeared to saturate (Bmax) at a molecular ratio of approximately 1.43 molecules of RNase H1 to 1 molecule of P32 suggesting that the RNase H1/P32 complex is formed at an equal molar ratio. Although efforts were made to reduce nonspecific binding of RNase H1 to the beads, the slightly higher than 1∶1 molecular ratio observed for RNase H1 was likely due to a small fraction of RNase H1 binding non-specifically to the beads. In addition, the interaction of human RNase H1 with P32 was ionic strength and pH dependent (Figure 2D). Specifically maximal binding was observed under NaCl concentrations less than 150 mM and binding was reduced at salt concentrations greater than 150 mM. Similarly, maximum binding was observed at pHs ranging from 6–8 with reduced binding above pH 8 (Figure 2D, lower Panel). These results suggest that RNase H1 and P32 bind optimally in physiological conditions.

### P32 Enhances the Turn-over and Reduces the binding affinity of RNase H1 for the substrate

To elucidate the functional significance of the interaction of P32 with human RNase H1, we determined the effects of P32 on the activity of RNase H1 by combining purified RNase H1 and GST-P32 at a molecular ratio of 1∶1 or 1∶5 with ^32^P labeled RNA/DNA duplex and determining the Michaelis-Menten kinetics and the binding affinity of RNase H1 for the hetero-duplex substrate. GST protein was used as a negative control (Figure 2E). The K_m_ and V_max_ were determined by measuring the initial cleavage rates for different concentrations of the heteroduplex (Figure 2E, upper panel) as previously shown for RNase H1 [40]. The binding affinity of RNase H1 for the substrate (K_d_) in the presence or absence of P32 was determined by inhibition analysis using an uncleavable 2′-fluroribonucleotide/DNA heteroduplex (Figure 2E, lower panel). An approximately 4 and 5-fold increase in K_m_ was observed for RNase H1 in the presence of, 1∶1 and 1∶5 ratio of P32 respectively, as compared to RNase H1 in the presence of the GST control protein. Consistent with the observed increase in K_m_, a similar increase in the dissociation constant (K_d_) was detected for the RNase H1/P32 complex binding to the heteroduplex substrate compared to RNase H1with the GST control protein, suggesting that P32 reduces the binding affinity of RNase H1 for the substrate. In addition, an approximately 2-fold increase in the k_cat_ was observed for the RNase H1/P32 complex compared to control RNase H1, resulting in a greater than 2-fold decrease in the bimolecular rate constant (k_cat_/K_m_). These results suggest that the enhanced cleavage rate observed for RNase H1 in the presence of P32 is likely not due to greater productive interactions between H1/P32 with the substrate. Instead, the enhanced turnover rate observed for the RNase H1/P32 complex is likely due to an increase in the off-rate between the RNase H1/P32 complex and the substrate. In addition, given that similar cleavage patterns were observed for RNase H1 in the presence or absence of P32, it is likely that P32 enhances the efficiency of RNase H1 without affecting the sequence preferences of the enzyme.

### P32 interacts with the hybrid binding domain of RNase H1

**Figure 3:**
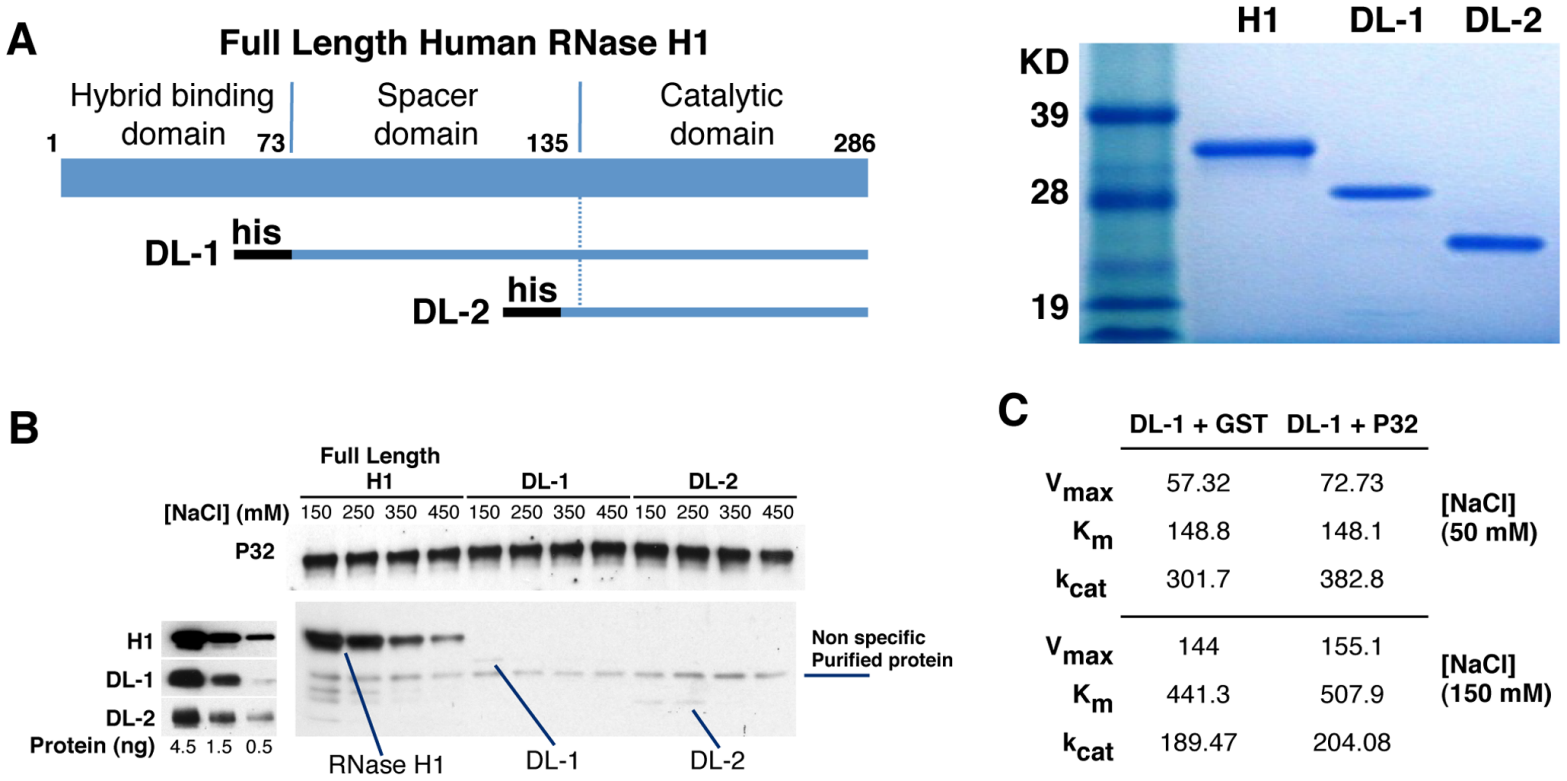
P32 appears to interact with the N-terminal duplex binding domain of RNase H1. (**A**) Expression and purification of RNase H1 deletion mutants. Left panel: Schematic depiction of the different human RNase H1 deletion mutants. DL1 deletes the hybrid binding domain (amino acid positions 1–73); DL2 deletes both the hybrid binding domain and the spacer domain (amino acid 1–129). The black bars at the N-terminus of each mutant represent a His tag. Right panel: Coomassie blue staining of the purified RNase H1 deletion mutants. The sizes of the standard markers are given. (**B**) Interaction of full length RNase H1 and its deletion mutants with P32. The full length or truncated RNase H1 proteins were incubated with GST-P32 bound to GST-beads under different NaCl concentrations ranging from 150–450 mM in both the binding and washing solutions. The P32 and RNase H1 or deletion mutants were eluted and analyzed by Western blot, using P32 or RNase H1 antibodies, respectively (right panel). Western blot to RNase H1 and deletion mutants DL1 and DL2 demonstrates that the mutant proteins are recognized by the RNase H1 antibody (left panel). (**C**) Michaelis-Menten Kinetics of DL-1 mutant in the presence or absence of P32. K_m_, V_max_, and k_cat_ for DL-1 plus GST or GST-P32 (DL-1:P32 = 1:5 in molecular ratio) were determined in 50 and 150 mM NaCl concentration with the Apo B RNA/DNA duplex as described in the Material and Methods.

To better characterize the interaction between human RNase H1 and P32 and to further understand which regions of the enzyme interact with P32, we expressed and purified two his-tagged RNase H1 deletion mutants (Figure 3A). RNase H1 is comprised of three functional domains: the N-terminal hybrid binding domain (aa1-72), spacer domain (aa73-135), and the C-terminal catalytic domain (aa136-286) (Figure 3A, left panel). The mutant DL1 deletes the hybrid binding domain and DL2 lacks both the hybrid binding domain and the spacer region leaving the catalytic domain only, which is similar to E. coli RNase H1. Both proteins have been previously shown to exhibit strong RNase H cleavage activity [17]. Neither the DL1 nor DL2 deletion mutant appeared to bind P32 in NaCl concentrations ranging from 150-450 mM (Figure 3B). These results suggest that P32 interacts with the hybrid binding domain of human RNase H1. Consistent with these results, P32 appeared to have little or no effect on the cleavage kinetics of the DL1 mutant for the RNA/DNA substrate (Figure 3C). The slight increase in the K_m_, V_max_ and k_cat_ observed for DL1 in the presence of P32 was insignificant compared to the effect of P32 on the cleavage kinetics of the full length RNase H1 (Figure 2E). Together, these results support the observation that P32 interacts with the N-terminal hybrid binding domain of human RNase.

### The RNase H1/P32 complex is involved in processing of mitochondrial pre 12/16S rRNA

**Figure 4:**
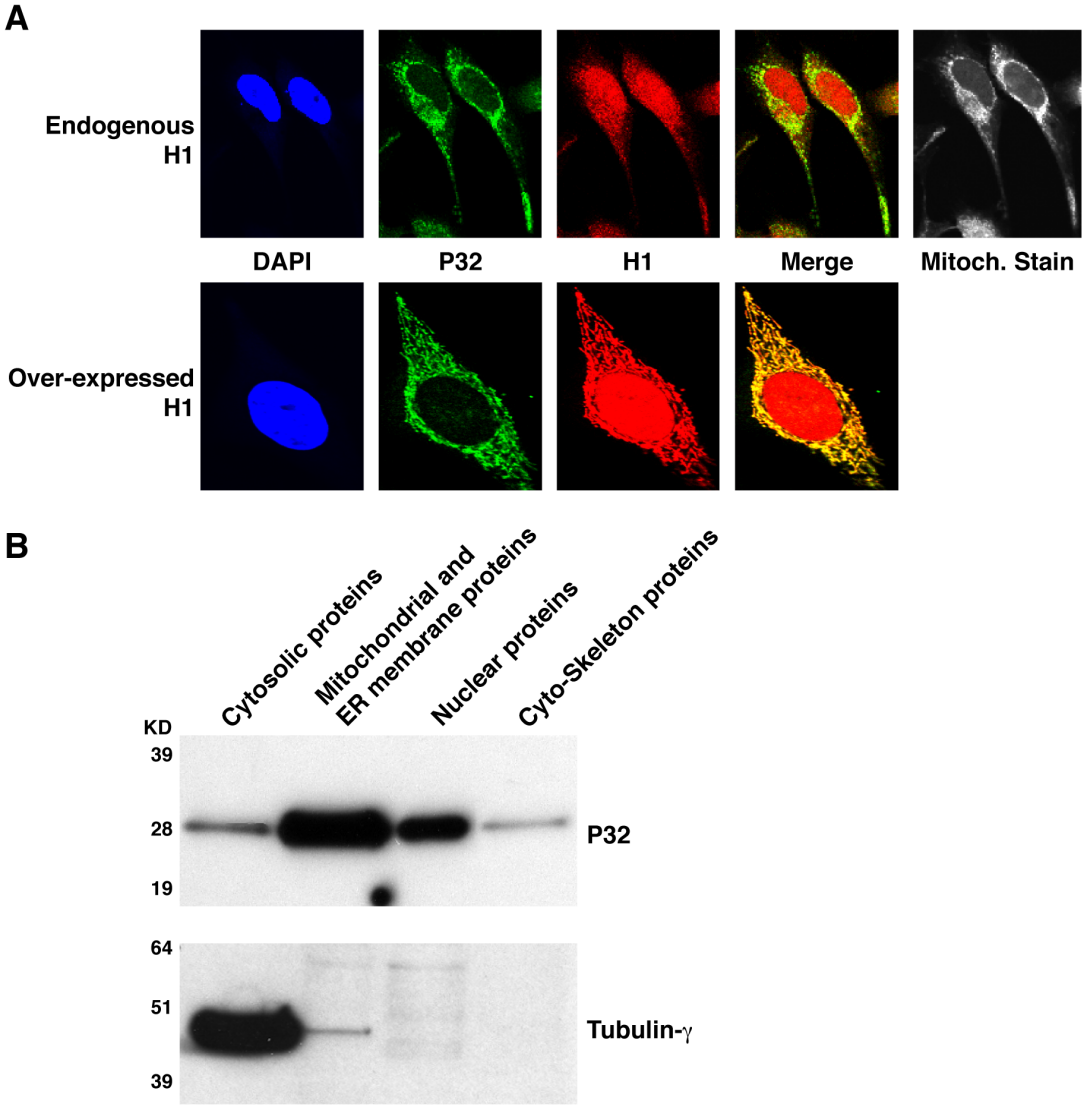
Co-localization of P32 and RNase H1. (**A**) Immunofluorescence Staining of P32 and RNase H1. Upper panel: HeLa cells were stained for endogenous P32 and RNase H1 using mouse monoclonal anti-P32 antibody and rabbit anti-RNase H1 antibody, respectively, followed by FITC conjugated donkey anti-mouse (**green**) and TRITC conjugated anti-rabbit secondary antibodies (**red**). Nuclei were stained with DAP1 (**Blue**) and Mitochondria were stained with mitotracker (**white**). Lower panel: HeLa cells were infected with adenovirus expressing RNase H1. Cells were stained as described in upper panel. (**B**) Subcellular fractionation of P32 protein. The proteins from sub-cellular compartments (cytosol, mitochondrial and ER membranes, nucleus and cytoskeleton) were prepared from HEK cells using proteome cell compartment kit (Qiagen). About 10 µg protein samples from each fraction were analyzed by western for P32. The same blot was stripped and tubulin-γ was detected to serve as a control.

It is known that human RNase H1 is present in both the cytoplasm and nucleus [40], and P32 is predominantly located in mitochondria but a fraction of this protein can also be detected in other subcellular locations including the nucleus [47]–[51]. We determined the cellular localization of the two proteins using immunofluorescent staining for endogenous P32 and RNase H1 in HeLa cells (Figure 4A). As expected, P32 is localized predominantly in the cytoplasm (Figure 4A upper panel) in a pattern consistent with a mitochondrial location. However, a small proportion of the P32 signal was also observed in the nucleus. The mitochondrial and nuclear locations of P32 were further confirmed by isolating nuclei and mitochondria and evaluating the levels of the protein via western analysis (Figure 4B). As expected, RNase H1 localized in both the nucleus and the cytoplasm and RNase H1 co-localized with P32 in mitochondria as determined by immunoflurescence staining (Figure 4A). To further confirm this co-localization, HeLa cells were infected with an adenovirus expressing full length RNase H1. The P32 and the over-expressed RNase H1 clearly co-localized in similar regions of cytoplasm, as determined with higher resolution imaging (Figure 4A, lower panel). The presence of a small amount of P32 in the nucleus suggests that P32 and RNase H1 may also co-localize in this compartment. However, since the highly enriched RNase H1 is evenly distributed in the nucleus, it is difficult to evaluate the subnuclear localization of overexpressed RNase H1.

**Figure 5:**
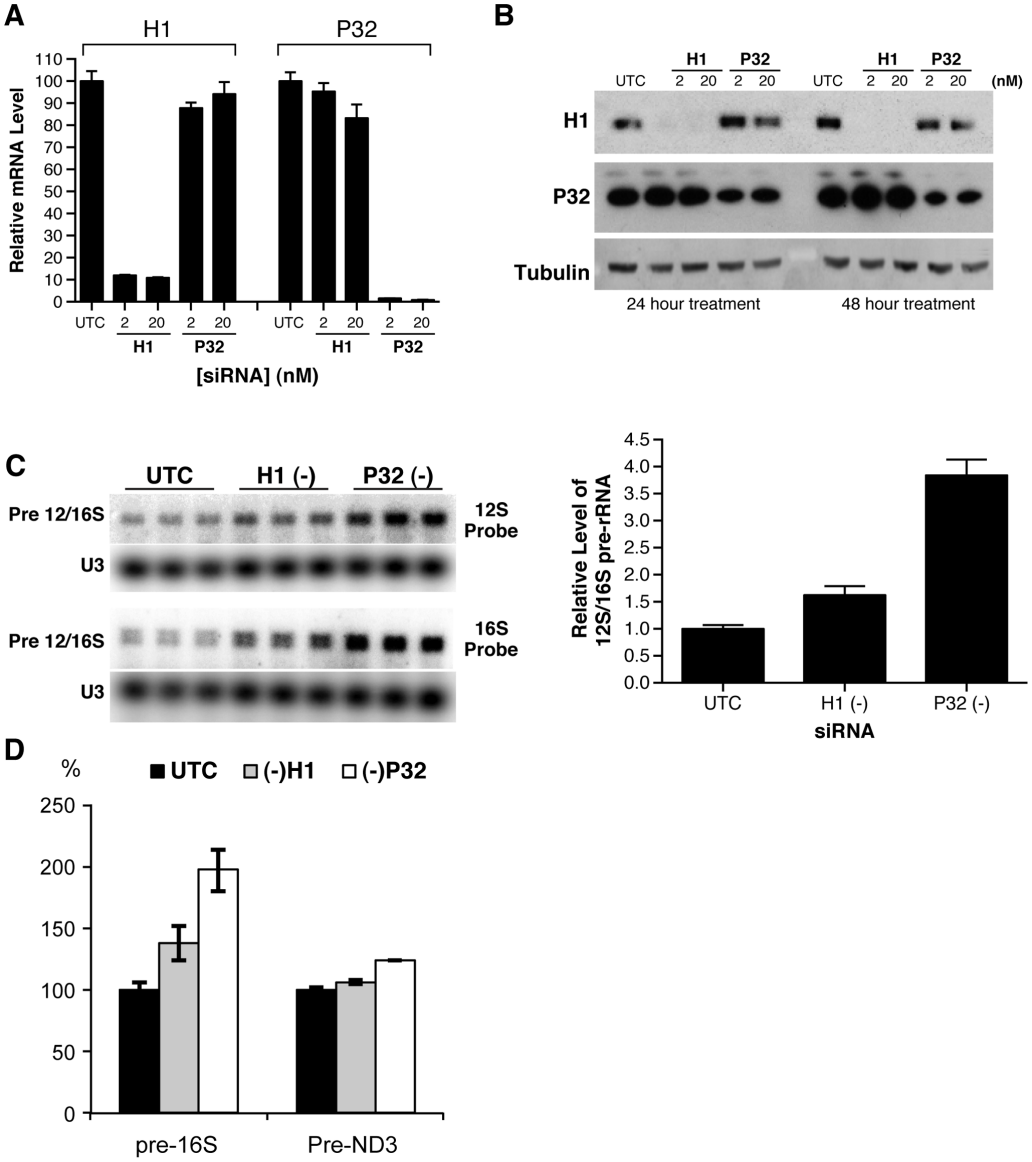
Depletion of RNase H1 or P32 resulted in accumulation of mitochondrial pre-12S/16S rRNA. HeLa cells were treated with 2 nM or 20 nM of RNase H1-siRNA or P32 –siRNA for 24 or 48 hours. (**A**) The mRNA levels of RNase H1 and P32 were determined by qRT-PCR 24 hrs after siRNA treatment. (**B**) Protein levels of RNase H1 and P32 were analyzed by western analysis 24 hours post siRNA treatment. (**C**) Reduction of RNase H1 or P32 significantly increased the level of mitochondrial pre-rRNA. HeLa cells were treated with either RNase H1-siRNA (2 nM) or P32-siRNA (2 nM) for 24 hours. Total RNA was prepared and subjected to Northern analysis with ^32^P labeled probes specific to 12S or 16S rRNAs. U3 snoRNA was detected and served as a control. The relative levels of pre-rRNA were measured from the results obtained with 12 S probe, normalized to U3, and plotted in the right panel. The error bars indicate standard error of the three replicates. (D) RT-PCR assay for the levels of pre-16 S and pre-ND3 RNAs. Total RNA prepared from HeLa cells treated for 24 hrs with corresponding siRNAs was analyzed by qRT-PCR, using primer probe sets specific to the tRNA^Val^-16 S rRNA junction (pre-16 S) or to the tRNA^Gly^-ND3 junction (pre-ND3). The error bars represent standard deviation of three replicates.

To investigate the potential roles of the RNase H1-P32 complex in mitochondrial RNA expression, we tested three sets of different siRNAs which specifically targeted either RNase H1 or P32. A greater than 90% reduction of RNase H1 or P32 mRNA was observed 24 hours after siRNA treatment (Figure 5A). The protein levels of RNase H1 and P32 were reduced by over 90% and 70%, respectively, after 48 hours of treatment (Figure 5B). Reduction of either RNase H1 or P32 mRNAs or proteins appeared to have no effect on the level of the other mRNAs or proteins evaluated (data not shown). In cells depleted of RNase H1, a significant increase in the 12 S/16 S mitochondrial rRNA precursor was observed, as demonstrated by northern hybridization using either a 12 S rRNA probe (Figure 5C, upper panel) or a 16 S rRNA probe (Figure 5C, middle panel). Reduction of RNase H1 increased the precursor level by more than 60% (Figure 5C, lower panel). Interestingly, an even greater increase (>300%) of the rRNA precursor was observed in P32 depleted cells, suggesting that reducing RNase H1 or P32 does not affect mtDNA transcription, however, subsequent RNA processing was impaired. This view is further supported by qRT-PCR results showing that reduction of RNase H1 or P32 caused accumulation of pre-16 S rRNA, whereas the level of the pre-ND3 region, which is part of the polycistronic transcript from the heavy strand, was not significantly affected (Figure 5D).

### RNase H1 and P32 Directly Interact with mtDNA and rRNA

**Figure 6:**
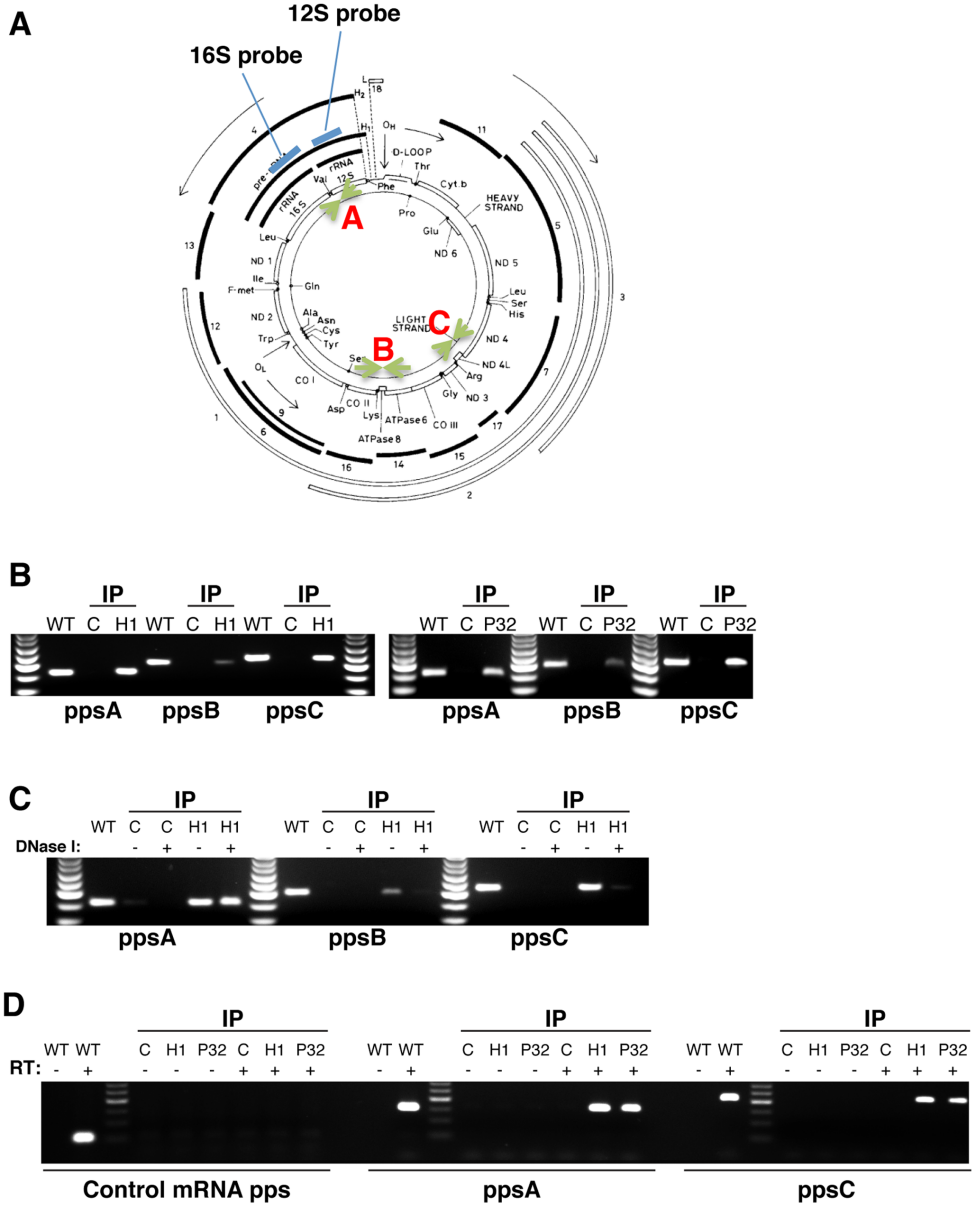
Both RNase H1 and P32 interact with mitochondrial DNA and pre-rRNA. (**A**) The positions of Probes and PCR primers for the human mitochondrial DNA. The DNA map was derived from published review [65]. Two oligonucleotide probes specific to 12 S and 16 S mitochondria rRNA regions are shown in **Blue bars**. Three sets of PCR probes corresponding to the A, B and C regions are indicated in **Green arrows**. (**B**) RNase H1 and P32 bind mitochondrial DNA. Cell extracts were prepared from an HA-H1 stably expressing cell line (RNase H1), control HEK cells or HEK cells transfected with the HA-P32 expression plasmid (P32). Equal amounts of each extract were used for immunoprecipitation with anti-HA beads. Nucleic acids were extracted from the precipitated samples using phenol/chloroform and subjected to PCR analysis. The probe sets for PCR were shown in Figure 6A. Genomic DNA from HEK cells that was used as a positive control. The PCR products were analyzed on 2% Agarose gels. (**C**) RNase H1 may interact with the mitochondrial rDNA region. The extracts from HA-H1 cell and control HEK cells were used for immunoprecipitation with HA-antibody. The precipitates were digested on beads with (+) or without (−) DNase I. The DNA associated with beads was then extracted and subjected to PCR analysis. The PCR products were separated in 2% agarose gel. (**D**) RNase H1 and P32 also co-immunoprecipitated with mitochondrial pre-rRNA. The same extracted nucleic acids from panel B were digested with DNase I. The RNA is used for reverse transcription with (+) or without (−) reverse transcriptase, followed by PCR amplification using different primer sets as indicated below the panels. PCR reaction using primers specific to U16 snoRNA was used as control.

Although the detrimental effects on mtRNA processing could be directly related to reduction of RNase H1 or P32, it is also possible that these observations are the result of secondary effects. To distinguish between these possibilities, immunoprecipitation followed by PCR analysis was employed to determine whether there was a direct interaction between these proteins and the mitochondrial DNA and/or the rRNA transcript. Lysates from the HA H1 stably transformed cells, HA P32 transiently expressing cells, and control cells were used for immunoprecipitation with the HA antibody. RNA and DNA were extracted from these immunoprecipitated samples and subjected to PCR amplification using three different PCR primer sets (pps) covering the 12 S/16 S rRNA region (pps A), ATPase 8/ATPase 6 region (pps B), and ND3/ND4 region (pps C) (Figure 6A). All three regions were successfully amplified by PCR from samples precipitated with either HA-RNase H1 or HA-P32, but not from samples precipitated from control cells without expression of the tagged proteins (Figure 6B), suggesting that mitochondrial DNA was specifically co-precipitated with RNase H1 and P32 and that these proteins are physically linked to mtDNA.

To identify the regions of mtDNA that interact with the RNase H1-P32 complex, the H1 immunoprecipitation beads were digested with DNase 1 to destroy the DNA regions that were not protected by proteins. The digested DNA was washed away, and the bead-associated DNA was prepared and analyzed by PCR amplification (Figure 6C). Clearly, DNase digestion abolished PCR amplification for primer sets B and C, but not set A, indicating that the mitochondrial DNA region encoding rRNAs (set A) is protected from DNase digestion and this region probably physically contacts RNase H1.

Next, we examined if pre-rRNA also physically interacts with the two proteins. Both HA-tagged RNase H1 and P32 were immunoprecipitated with anti-HA beads. Nucleic acids were prepared from the precipitated samples and subjected to DNase I digestion. DNA-removed RNA was extracted and reverse transcription was performed to synthesize cDNA. PCR was performed with cDNA or RNA samples without reverse transcription. Region A was amplified by PCR from RNase H1 or P32 precipitated materials only after reverse-transcription (Figure 6D), suggesting that precursor rRNA was co-precipitated with these two proteins. Note that RNA transcripts covering region C were also precipitated with these two proteins. This is not surprising, since the mitochondrial DNA is transcribed into long polycistronic molecules which can be detected by reverse-transcription PCR. On the other hand, H1 and P32 may interact with many RNA regions of the polycistronic molecule. Regardless of this uncertainty, it is clear that RNase H1 and P32 physically interact with the mitochondria precursor RNAs.

### P32 physically interacts with the mitochondrial RNase P protein MRPP1

**Figure 7:**
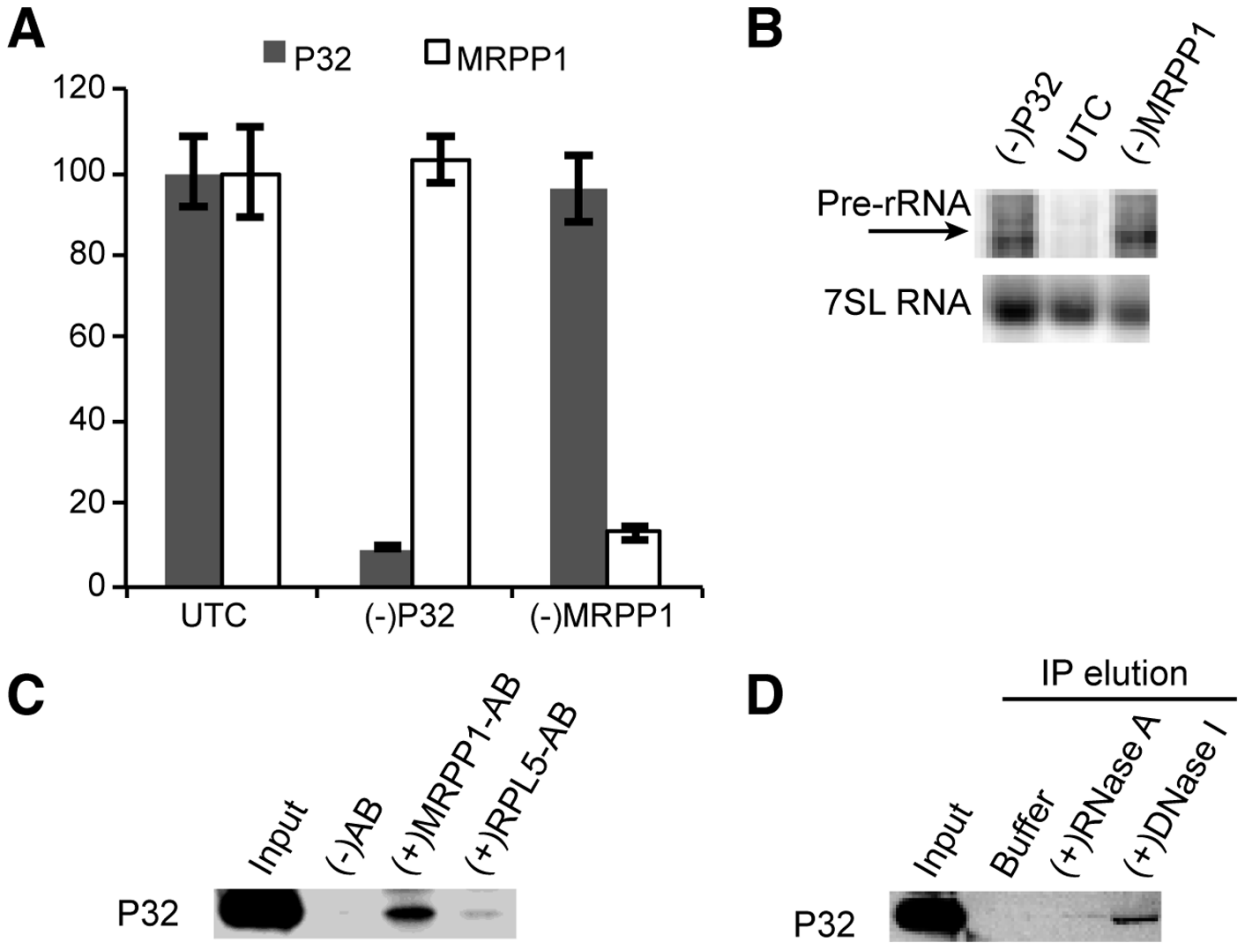
P32 can be co-immunoprecipitated with mitochondrial RNase P protein. (**A**) RT-PCR assay for the mRNA levels of P32 or MRPP1 in siRNA treated HeLa cells. The error bars represent standard deviation of three replicates. (**B**) Northern hybridization for mitochondrial pre-rRNA. Total RNA prepared from HeLa cells pre-treated for 24 hrs with P32 [(−)P32] or MRPP1 [(−)MRPP1] siRNAs was analyzed by northern hybridization, using probes specific to pre-rRNA, as in Figure 5.The same blot was re-probed for 7 SL RNA, which served as a loading control. (C) Immunoprecipitation using MRPP1 antibody (MRPP1-AB) or RPL5 antibody (RPL5-AB) was performed as described in materials and methods. Co-isolated proteins were separated by SDS-PAGE, and P32 protein was determined by western analysis. (−)AB, control immunoprecipitation in the absence of antibody. (D) DNase I treatment disrupted the P32-MRPP1 interaction. Immunoprecipitation was performed using MRPP1 antibody, as in panel C. After wash, the beads were incubated with either RNase A or DNase I, to elute bound materials. The eluted proteins were analyzed by western blots for the presence of P32 protein. Buffer, 1×TE buffer alone.

Since P32 itself does not contain nuclease domains and RNase H1 acts on DNA/RNA duplexes, the involvement of P32 and RNase H1 in mitochondria pre-rRNA processing may be mediated by other factors required for mitochondrial RNA processing. It is known that mitochondrial pre-rRNA processing is preceded by processing of tRNAs and this is mediated by mitochondrial RNase P, a protein complex that is composed of three subunits (MRPP1, MRPP2, and MRPP3) and is responsible for tRNA 5′ processing [52], [53]. Consistent with previous reports [53], siRNA-mediated reduction of MRPP1 led to accumulation of mitochondrial pre-rRNA, similar to the effects observed for P32 or RNase H1 reduction, as determined by northern hybridization (Figure 7A and 7B). Importantly, P32 was co-precipitated with MRPP1, but not with a control protein, RPL5, as demonstrated by immunoprecipitation followed by western analysis (Figure 7C), suggesting a physical link between P32 and the RNase P complex. Although we failed to detect co-precipitation of RNase H1 with MRPP1, even for RNase H1 over-expressed cells (data not shown), we cannot rule out the possibility that RNase H1 may also be linked to the RNase P complex by P32, given that the RNase H1 expression level is very low, or the interaction, if any, may be transient in nature.

To determine whether P32 and MRPP1 interact directly through a protein-protein interaction or if the interaction is mediated by DNA or RNA, immunoprecipitation was performed again using the MRPP1 antibody, and the washed beads containing co-precipitated materials were digested by either DNase I or RNase A. The nuclease released materials were analyzed by western blots. As shown in Figure 7D, a significant amount of P32 was released from the beads by DNase I treatment, but not by RNase A treatment, suggesting that the physical interaction between P32 and MRPP1 may be mediated by DNA. Together, these results suggest that the P32/H1 complex is linked to the RNase P complex, most likely by binding to mitochondrial DNA and/or nascent RNA transcripts.

## Discussion

In this study, we show that human RNase H1 but not H2 is associated with the mitochondrial protein P32. P32 appears to bind to the hybrid binding domain of RNase H1 and significantly enhances the cleavage activity of the enzyme. Results from the gel shift experiment indicate that only purified RNase H1 and not P32 is able to directly bind the RNA/DNA duplex (Figure 2B). Our results clearly show that P32 enhances the efficiency of the RNase H1 without affecting the cleavage specificity of the enzyme. Such an enhancement is most likely achieved by reducing the binding affinity of the enzyme to the substrate and enhancing the turnover rate for the RNase H1/P32 complex via an increased off-rate (Figure 2E). Thus, it is possible that binding of P32 to RNase H1 alters the binding characteristics of RNase H1, leading to a weaker interaction of RNase H1 with the nucleic acid substrate. Finally, this enhancement requires the presence of the hybrid binding domain of RNase H1 (Figure 3), suggesting a direct physical interaction between P32 and RNase H1.

The hybrid binding domain has been shown to contribute to the binding affinity of RNase H1 for the substrate through both electrostatic and stacking interactions. Therefore, a possible explanation for the observed reduction in the binding affinity of RNase H1 for the heteroduplex in the presence of P32 is that the interaction between P32 and the hybrid binding domain may interfere with the ability of the hybrid binding domain to bind the substrate. However, given that the hybrid binding domain has also been shown to be responsible for the strong positional preference for cleavage observed for human RNase H1 which was not exhibited by either the hybrid binding domain deletion mutants of the human enzyme or the E. coli enzyme that does not contain the hybrid binding domain [15], [20], the similarities between the cleavage patterns for human RNase H1 in the presence or absence of P32 suggest that it is more likely that in the presence of P32, the hybrid binding domain undergoes conformational changes that reduce the affinity for substrate without altering the basic characteristics of binding.

This observation is interesting from an evolutionary prospective as well. Compared to the E. coli RNase H1, the addition of the RNA binding and spacer domains of human RNase H1 appears to sacrifice enzymatic efficiency for cleavage site specificity. Here we show that P32 binds human RNase H1 and recovers most the enzymological efficiency while maintaining the cleavage specificity. In addition, we have previously shown that the catalytic domain of human RNase H1 contains vicinal cysteines that are not found in the E. coli enzyme and make the human enzyme very susceptible to oxidation that inactivates the enzyme [43]. We have referred to this as a ‘redox switch’. It is tempting to speculate that the ‘redox switch’ and the interaction of P32 with the hybrid binding domain provide two mechanisms by which the activity of human RNase H1 may be regulated. Although there are a good many examples of nucleases that are inactive as monomers and require protein cofactors, e.g. RNase H2, to our knowledge our paper is the first to show that a nuclease that is active as a monomer has its basic enzymological properties modified by another protein.

The diverse functions reported for P32 suggest that the P32 may be a multifunctional chaperone protein [54], [55]. It is a 32Kd acidic protein localized predominantly in mitochondrial matrix, but has been reported to be present in other subcellular locations including the nucleus and the surface of tumor cells [47]–[49] [48], [56]. Both P32 and RNase H1 co-localize in the mitochondrial compartment and perhaps the nucleus as well (Figure 4).

RNase H1 and P32 have been previously implicated in different biological processes in mitochondria [15], [57]. Co-localization of RNase H1 and P32 in this cellular compartment suggests that the two proteins may be involved in the same or similar biological processes in mitochondria. RNase H1 was shown to be involved in mitochondrial DNA (mtDNA) replication and is able to remove the R-loops formed between the DNA template and nascent RNAs [37], [58], [59]. In addition, it has been documented that transcription of mitochondrial genes is directly linked to replication [60) and in many cases coupling of nuclear RNA processing and transcription (e.g. splicing and nuclear rRNA processing) has been reported [61], [62], suggesting a potential influence of RNase H1 on mtRNA expression. In addition, P32 was already shown to be involved in nuclear rRNA maturation as depletion of P32 by siRNA treatment caused reduction of mature rRNAs and accumulation of a pre-rRNA species [63]. Thus, it is also possible that RNase H1 and/or P32 may play a role in expression of mtRNAs, including the mitochondria rRNAs. Interestingly and surprisingly, we found that siRNA-mediated reduction of either RNase H1 or P32 caused significant accumulation of mitochondrial 12 S/16 S precursor rRNA. Although we cannot completely exclude the possibility that such effects are secondary to depletion of these proteins, it is more likely and we favor the possibility that this is a direct effect since RNase H1 and P32 physically contact mtDNA and the polycistronic RNA transcript, as demonstrated using the immunoprecipitation assay.

That P32 may play a role in RNA processing has been proposed previously as it was initially identified as a splicing factor [64]. In addition, a recent study reported that P32 is involved in nuclear pre-rRNA processing, by regulating the binding of other proteins to pre-rRNA [63]. More relevant to this study is a most recent finding showing that P32 protein associates with mitochondria rRNA and is involved in mitochondria ribosome formation [57]. It was found that siRNA mediated depletion of P32 led to reduced levels of 55S mature ribosome, yet the levels of both ribosome subunits were not affected. These observations are consistent with our findings that reduction of P32 (and H1) caused accumulation of the pre-rRNA species, whereas the levels of mature mitochondria rRNAs were not significantly affected (data not shown), suggesting a slower biogenesis of the mitochondria ribosome.

Currently it is not clear how RNase H1 and P32 are involved in mtRNA maturation. Our immunoprecipitation results showed that at least P32 physically interacts with the mitochondrial RNase P complex, which is required for mitochondrial RNA processing [53]. P32 (and perhaps also RNase H1) may affect pre-rRNA processing by modulating the function of the RNase P complex, which is responsible for the 5′ processing of tRNAs from the polycistronic transcripts, an event required for the release and maturation of rRNAs and other protein coding RNAs from the polycistronic molecule. It is possible that such an effect is mediated by the potential coupling between transcription and RNA processing, as is the case in the nucleus where transcription and RNA processing are coupled [61], [62]. The linkage between P32 and MRPP1 is mediated by DNA, suggests that a co-transcriptional effect of P32/H1 on pre-rRNA processing is also possible. In addition, It has been shown that RNase H1 is required for removal of the RNA strand within R-loops formed during transcription [59]. Thus, it is possible that RNase H1 and/or P32 may act on the nascent transcript to affect its processing, either directly or mediated by other components. Further understanding of the underlying mechanisms of RNase H1/P32-involved mitochondrial rRNA maturation will be an interesting challenge for future studies.
